# Characterization of a New *Pseudomonas Putida* Strain Ch2, a Degrader of Toxic Anthropogenic Compounds *Epsilon*-Caprolactam and Glyphosate

**DOI:** 10.3390/microorganisms11030650

**Published:** 2023-03-03

**Authors:** Tatiana Z. Esikova, Tatiana O. Anokhina, Nataliya E. Suzina, Tatiana V. Shushkova, Yonghong Wu, Inna P. Solyanikova

**Affiliations:** 1 Laboratory of Plasmid Biology, G.K. Skryabin Institute of Biochemistry and Physiology of Microorganisms, Pushchino Center for Biological Research of the Russian Academy of Sciences, Prosp. Nauki 5, Pushchino, 142290 Pushchino, Russia; 2 Laboratory of Cytology of Microorganisms, G.K. Skryabin Institute of Biochemistry and Physiology of Microorganisms, Pushchino Scientific Center for Biological Research of the Russian Academy of Sciences, Prosp. Nauki 5, Pushchino, 142290 Pushchino, Russia; 3 Laboratory of Microbial Enzymology, G.K. Skryabin Institute of Biochemistry and Physiology of Microorganisms, Pushchino Center for Biological Research of the Russian Academy of Sciences, Prosp. Nauki 5, Pushchino, 142290 Pushchino, Russia; 4 Zigui Ecological Station for Three Gorges Dam Project, State Key Laboratory of Soil and Sustainable Agriculture, Institute of Soil Science, Chinese Academy of Sciences, 71 East Beijing Road, Nanjing 210008, China; 5 Regional Microbiological Center, Institute of Pharmacy, Chemistry and Biology, Belgorod National Research University, 308015 Belgorod, Russia

**Keywords:** *Pseudomonas putida*, biodegradation plasmids, *epsilon*-caprolactam, glyphosate, salicylate, polyhydroxyalkanoates, ultrastructural organization of cells

## Abstract

In this work, a new Ch2 strain was isolated from soils polluted by agrochemical production wastes. This strain has a unique ability to utilize toxic synthetic compounds such as *epsilon*-caprolactam (CAP) as a sole carbon and energy source and the herbicide glyphosate (GP) as a sole source of phosphorus. Analysis of the nucleotide sequence of the 16S rRNA gene of Ch2 revealed that the strain belongs to the species *Pseudomonas putida*. This strain grew in the mineral medium containing CAP in a concentration range of 0.5 to 5.0 g/L and utilized 6-aminohexanoic acid and adipic acid, which are the intermediate products of CAP catabolism. The ability of strain Ch2 to degrade CAP is determined by a conjugative megaplasmid that is 550 kb in size. When strain Ch2 is cultured in a mineral medium containing GP (500 mg/L), more intensive utilization of the herbicide occurs in the phase of active growth. In the phase of declining growth, there is an accumulation of aminomethylphosphonic acid, which indicates that the C-N bond is the first site cleaved during GP degradation (glyphosate oxidoreductase pathway). Culture growth in the presence of GP during the early step of its degradation is accompanied by unique substrate-dependent changes in the cytoplasm, including the formation of vesicles of cytoplasmic membrane consisting of specific electron-dense content. There is a debate about whether these membrane formations are analogous to metabolosomes, where the primary degradation of the herbicide can take place. The studied strain is notable for its ability to produce polyhydroxyalkanoates (PHAs) when grown in mineral medium containing GP. At the beginning of the stationary growth phase, it was shown that, the amount and size of PHA inclusions in the cells drastically increased; they filled almost the entire volume of cell cytoplasm. The obtained results show that the strain *P. putida* Ch2 can be successfully used for the PHAs’ production. Moreover, the ability of *P. putida* Ch2 to degrade CAP and GP determines the prospects of its application for the biological cleanup of CAP production wastes and in situ bioremediation of soil polluted with GP.

## 1. Introduction

One of the most important problems today is environmental pollution by persistent toxic substances. The main sources of pollution are synthetic chemicals produced by human activity that are released into the environment because of their widespread use, as well as agro-industrial and household wastes, crude oil and petroleum refinery products. Organic pollutants are highly toxic, resistant to degradation under natural conditions, have poor water solubility and can accumulate in living organisms [1]. Due to their toxicity, mutagenic and carcinogenic properties, the pollutants can cause harmful effects on living organisms.

Microorganisms from various taxonomic groups can utilize a large number of organic substances both natural and anthropogenic origins. For several decades, numerous studies have been conducted to show bacteria belonging to the genus *Pseudomonas* are well known for the ability to degrade toxic pollutants. The genus *Pseudomonas* represents a large taxonomic group of bacterial species (309 valid species as of February 2023 [2]) that show great metabolic, physiological and genetic diversity [3]. Pseudomonads are widely distributed in nature and inhabit a wide range of niches, like uncontaminated and contaminated soil, water and rhizospheres of plants. Among species belonging to the genus *Pseudomonas*, the representatives of *P. putida* have an outstanding ability to biodegrade xenobiotics; this is due to the peculiarities of their genetic organization [4]. For instance, the well-known *P. putida* strain KT2440 used more than 100 studied complex organic compounds as the sole carbon source [5]. Analysis of the whole genome of *P. putida* KT2440 (6.18 Mbp) revealed the genes encoding many oxygenases, oxidoreductases, dehydrogenases, monooxygenases, dioxygenases, hydrolases and so forth. The combined action of these enzymes belonging to different protein families promotes exceptional metabolic versatility of *P. putida* KT2440.

To date, many *P. putida* strains have been described for the ability to utilize both natural and anthropogenic pollutants, including alkanes [6], substituted and unsubstituted aromatic hydrocarbons [7], dyes [8], solvents [9], herbicides [10], insecticides [11], cyclic amides and nylon oligomers [12] and so forth. In many cases, it was shown that the catabolic genes are localized on plasmids [6,7,11,12]. Biodegradation plasmids, due to their ability to conjugate transfer, as well as the presence of transposons and genomic islands in their composition, play a leading role in dissemination of catabolic genes in microbial communities and their adaptation to changing environmental conditions [13].

It Is also worthy of note that bacteria from the genus *Pseudomonas* are extremely attractive not only for the implementation of pollutant biotransformation processes but also for the production of bioactive metabolites, lipids, proteins and biopolymers [14]. For instance, *P. putida* strains have been described for their ability to accumulate polyhydroxyalkanoates (PHAs), the polymers of hydroxy-fatty acid derivatives [15]. PHAs are a valuable material in biotechnological production, since they are considered as an alternative to nondegradable synthetic plastic. Strains capable of synthesizing PHAs while growing on wastes from oil-chemical and chemical manufacturers may be of particular interest [16,17,18]. PHA production costs and environmental waste can be decreased by using such readily available and inexpensive chemicals as growth substrates for bacteria that produce PHAs. 

*Epsilon*-caprolactam (caprolactam, CAP) (cyclic amide (lactam) of 6-aminohexanoic acid) is one of the most in-demand chemicals on the global market. CAP is used in the manufacture of a nylon-6 polymer. It is a toxic compound that possesses high mutagenic activity and induces chromosome aberrations in mammals [19]. Moreover, this xenobiotic compound can also inhibit the growth of plants and microorganisms that play a key role in maintaining the ecological balance of soils [20]. Nowadays, wastes from CAP production are burned or buried, leading to pollution of soils and underground water [21]. 

Glyphosate (N-(phosphonomethyl)glycine) (GP) is a broad-spectrum, non-selective herbicide that is widely used in agriculture for the control of weedy species. Its accumulation in soils and penetration into water reservoirs causes alterations in soil and water microflora and harms the health of animals and humans [22]. Ecological risks are also driven by aminomethylphosphonic acid (AMPA), the primary intermediate of GP biodegradation. AMPA is more persistent than GP and slowly biodegradable; it exhibits toxic and mutagenic effects on microorganisms, plants and animals [23,24,25]. As with CAP, microbial biodegradation depends on the presence of specific enzyme systems and is the most effective way to remove GP from the environment [26,27,28,29]. That is why the study of the peculiarities of the degradation of CAP and GP by bacterial strains is an urgent and promising task.

The aim of this research was to investigate a *Pseudomonas putida* Ch2 strain capable of utilizing several organic compounds including toxic anthropogenic chemicals like CAP and GP. A special focus was on the harmful effects of these chemical compounds on ultrathin cell structure and the degradative ability of the strain under study.

## 2. Material and Methods

### 2.1. Chemicals

For the preparation of culture media, the reagents used were obtained from the following companies: Reachim (Moscow, Russia), AppliChem (Darmstadt, Germany) and Panreac (Barcelona, Spain). Biochemical reagents were obtained from Sigma-Aldrich (Sigma, St. Louis, MO, USA), Serva (Heidelberg, Germany) and Fluka (Seelze, Germany), and molecular biology reagents were obtained from Thermo Fischer Scientific (Waltham, MA, USA), Zymo Research (Irvine, CA, USA), New England BioLabs (Hitchin, UK) and Biocom (Moscow, Russia).

### 2.2. Isolation of Bacteria and Cultivation Conditions

Soil samples were taken from the site of an agrochemical enterprise (Voskresenskoye, Nizhy Novgorod region, Russia). The direct plating method was used to isolate bacteria. For this purpose, soil samples (5.0 g) were suspended in 45 mL of sterile 0.85% (*w/v*) NaCl solution and incubated at 28 °C, 180 rpm for 2 h. Ten-fold dilutions of the soil suspensions were spread over plates containing Luria Bertani (LB) growth media [30]. Plates were incubated at 28 °C for 3 days. Colonies with different cultural and morphological characteristics were selected for further investigation. The purity of the isolated cultures was controlled by light microscopy (Nikon Eclipse Ci microscope, (Nikon, Tokyo, Japan) equipped with ProgRes SpeedXT camera (Jenoptic, Jena, Germany)).

### 2.3. Investigation of the Ability of Isolates to Utilize Different Organic Substrates

The selected bacterial strains were tested for the ability to utilize different aromatic, aliphatic and chlorinated compounds as a sole sources of carbon and energy. The bacteria were cultured in M9 mineral medium [30] containing a specific substrate. After autoclaving, a sterile substrate was added to M9 medium. The concentrations of substrates used were as follows: 0.5 g/L salicylate, gentisate, protocatechuate, ortho-phthalate and benzoate; 0.2 g/L phenol, chlorobenzoates (2-, 3-, 4-chlorobenzoate) and 2,4-dichlorophenoxyacetic acid; 0.1 g/L 2,4-dichlorophenol and 2,4,6-trichlorophenol; and 1.0 g/L caprolactam. 

Water-insoluble aromatic acids were added into the growth medium as sodium salts. When microorganisms were grown on an agarized medium, the lid’s inner surface of an inverted Petri dish was coated with volatile aromatic and aliphatic compounds such as naphthalene, toluene, *para*-xylene, ethylbenzene, hexane, octane, hexadecane and diesel oil. Bacteria were cultured on selective media at 28 °C and the evaluation of bacterial growth was made within an interval of 3–7 days. 

Growth of isolates used GP as a sole source of phosphorus was studied in mineral medium MS1 with a composition of (g/L): NH_4_Cl–2.0, MgSO_4_·7H_2_O–0.2 and K_2_SO_4_–0.5; and microelements (mg/L) FeSO_4_·7H_2_O–2.5, CaCl_2_·6H_2_O–10.0, CuSO_4_·5H_2_O–2.0, H_3_BO_3_–0.06, ZnSO_4_·7H_2_O–20.0, MnSO_4_·H_2_O–1.0, NiCl_2_·6H_2_O–0.05 and Na_2_MoO_4_·2H_2_O–0.3, pH 7.0–7.2. GP (500 mg/L) were added to MS1 in the form of GroundBio herbicide (36% solution of isopropyl amine salt of glyphosate, Technoexport, Moscow, Russia) that was similar in composition to Roundup herbicide (Roundup, Monsanto, Saint Louis, MO, USA). Sodium glutamate (1.0%, *w*/*v*) was used as a carbon source [31].

### 2.4. Study of Growth Dynamics of Ch2 Strain in Mineral Medium with Caprolactam and Its Intermediates 

To study the dynamics of growth of Ch2 strain in the medium containing CAP and its intermediates 6-aminohexanoic acid (6-AHA) and adipic acid (ADA) as sole sources of carbon and energy, M9 liquid mineral medium was used. Cells were cultured in 750 mL Erlenmeyer flasks with 100 mL of the medium at 24 °C and 180 rpm. The substrate concentration was 1.0 g/L (*w*/*v*). Microbial growth was assessed by measuring the optical density at the wavelength of 560 nm using a Shimadzu UV-1800 spectrophotometer (Kyoto, Japan). Culture grown on the same medium up to the mid-exponential growth phase was used as an inoculum. Cells were concentrated by centrifugation (Rotanta 460R, Nettetal, Germany) at 5000 g and 4 °C for 10 min, resuspended in a small amount of sterile M9 medium and then transferred into flasks to fit the initial optical density range of 0.06–0.08. 

Growth of Ch2 at different CAP concentrations was examined in an M9-containing substrate at 0.4, 0.5, 1.0, 2.0, 5.0, 7.5, 10.0 and 15.0 g/L. The conditions for the preparation of inoculum and cultivation of cells were the same.

### 2.5. A Study of the Dynamics of Glyphosate Degradation by Ch2 Strain

To study the dynamics of GP consumption, herbicide and glutamate were added into MS1 liquid medium as described above [31]. To induce degradative enzymes, cells were grown on MS1 agar medium supplemented with GP and glutamate for 3 days and then washed off with MS1 and transferred into the flask with carbon source but without phosphorus (phosphorus starvation) and incubated on a shaker for 48 h. Then, an inoculum prepared in such manner was added to a fresh medium to fit the initial optical density range of 0.2–0.3. Cells were cultured in 750 mL Erlenmeyer flasks containing 100 mL of the medium on a shaker (180 rpm) at 28 °C. During cultivation, the solution of H_2_SO_4_ (20%, *v/v*) was added to maintain the pH of the medium within the range of 7.0–7.5. Probes for analysis were taken up every 24 h for 14 days. Culture growth was monitored by measuring changes in the optical density with a Shimadzu UV-1800 spectrophotometer (Kyoto, Japan) at the wavelength of 560 nm (OD560), GP was determined by high-performance liquid chromatography (HPLC) [32].

### 2.6. Analytical Methods for Characterization of the Glyphosate Degradation Process

#### 2.6.1. High-Performance Liquid Chromatography

Glyphosate was determined in culture liquid by HPLC (Sykam chromatography system, Eresing, Germany) with UV detection at 206 nm, Repro-Gel H column, 9 mkm, 250 × 8 mm (Dr. Maisch, Ammerbuch, Germany) at 65 °C. The mobile phase was 0.01 N H_2_SO_4_, elution rate of 1 mL/min [31]. 

#### 2.6.2. Thin-Layer Chromatography

The mobility of dansyl derivatized glyphosate, its transformation products, and glutamic acid was investigated using the method described previously [33]. A solution of dansyl chloride in acetone (6 mg/mL) was used as the reagent. To convert amino acids and phosphonic acid amides of filtrates of culture liquids into dansyl derivatives, 90 μL of a biological sample was mixed with 10 μL of 1 M NaHCO_3_ and 100 μL of the reagent, and the mixture was left at 24 °C overnight in the dark. 

The chromatographic mobility of the dansyl derivatives of glyphosate, the products of its transformation, and also glutamic acid were examined using Sorbfil PTSH-P-V plates (ZAO Sorbpolimer, Krasnodar, Russia). The development length was 7.5 cm and the temperature was in the range of 21–23 °C. On the plates, 2–10 μL of samples were applied. Visualization was performed using a UFS 254/365 chromatographic lamp (ZAO Sorbpolimer, Krasnodar, Russia) at a working wavelength of 365 nm.

One-dimensional chromatography with the subsequent use of two mobile phases appeared to be the most efficient for the identification of dansyl derivatives of phosphonic acids. The plates were first developed in the weakly acidic mobile phase chloroform–methanol–acetic acid (25:5:0.2, *v*/*v*/*v*). After the silica was dried in the flow of air, the plate was developed again in the basic system ethanol–24% ammonium hydroxide solution (Sigma-Aldrich, Burlington, MA, USA) in the ratio 7:3 (*v*/*v*).

### 2.7. Molecular Genetic Methods

#### 2.7.1. The 16S rRNA Gene Sequencing and Phylogenetic Analysis

The genomic DNA was extracted using Zymo Researcher Quick-DNA Fungal/Bacterial Miniprep Kit (Zymo Research, Irvine, CA, USA) following the manufacturer’s recommendations. Amplification of the 16S rRNA gene was performed with the GeneAmp PCR System 9700 with prokaryotic universal 16S rRNA gene primers: 27f (5′-AGAGTTTGATCCTGGCTCAG-3′) and 1492r (50-TACGGYTACCTTGTTACGACTT-30) [34]. Isolation and purification of PCR products were carried out using ZymoClean Gel DNA Recovery Kit (Zymo Research, Irvine, CA, USA) according to the instructions of the manufacturer. DNA sequencing was performed on an automated ABI Prism 373 3130XL sequencer (Applied Biosystems, Waltham, MA, USA).

A preliminary phylogenetic analysis of the determined nucleotide sequence was performed using the BLAST software package and GenBank database [35]. To determine more precisely the phylogenetic position of the studied strain, its 16S rRNA gene sequence was aligned with the corresponding sequences from microorganisms of the closely related bacteria using the CLUSTAL W software package [36]. 

#### 2.7.2. Conjugal Transfer of Plasmids 

A conjugal transfer of plasmids was carried out according to Dunn and Gunsalus method [37]. Cells of donor and recipient strains in a logarithmic growth phase were mixed in a ratio of 1:2 (*v*/*v*), then cell mixture was spread on LB plates and incubated for 15–18 h at 28 °C. Further, cells were washed off with sterile solution of 0.85% (*w*/*v*) NaCl and placed on Petri dishes with M9 selective medium containing caprolactam or salicylate at a concentration of 1.0 g/L as the carbon source and the antibiotic kanamycin at a concentration of 100 µg/mL. *P. putida* strain KT2442 was used as a recipient strain (Gfp^+^, Km^r^, Rif^r^) [38].

#### 2.7.3. Pulsed-Field Gel Electrophoresis of the Genomic DNA

To visualize plasmid DNA, pulsed-field gel electrophoresis of the genomic DNA was carried out [39]. Electrophoresis was performed in pulsed fields using a hexagonal electrode on the electrophoresis apparatus from Pharmacia-LKB (Boston, MA, USA) following the manufacturer’s instructions.

### 2.8. Microscopy

Light microscopy of samples in the phase contrast mode was carried out using a Nikon Eclipse Ci microscope (Nikon, Tokyo, Japan) equipped with a ProgRes SpeedXT camera (Jenoptic, Jena, Germany). Electron microscopy of thin sections was carried out according to the method described in [40].

To prepare ultrathin sections, double fixation with glutaraldehyde and osmium tetraoxide was used followed by embedding the cells in an epoxy resin Epon 812. Sections were viewed through an electron microscope JEM-1400 (JEOL, Tokyo, Japan) at an accelerating voltage of 80 кV.

### 2.9. Statistical Data Processing

The mean values and standard deviations were calculated based on data obtained from three independent experiments using Microsoft Excel 2007 [41].

## 3. Results and Discussion

### 3.1. Isolation of Ch2 Strain and Determination of Its Ability to Utilize Various Pollutants

From the soil samples collected at the site of agrochemicals enterprise, 20 pure bacterial cultures with different colonies morphology have been isolated by direct spreading onto LB medium. The isolated bacterial strains were tested for the ability to utilize different organic compounds such as aromatic, aliphatic, chlorine-containing compounds, caprolactam and glyphosate. As a result, most of the isolates did not grow in the medium supplemented with the above mentioned substrates or utilized only one of these substrates. Strain Ch2 (VKM B-3631D) was chosen for further investigations, as it was able to utilize benzoate, protocatechuate, salicylate and caprolactam as sole sources of carbon and energy and glyphosate as a source of phosphorus. To the best of our knowledge, a unique ability of the bacterium to degrade simultaneously these natural aromatic compounds and anthropogenic products has not been previously described.

It is known that phenolic compounds (including protocatechuic, salicylic and *p*-hydroxybenzoic acids) are present in plants and play an essential role in physiological processes like photosynthesis, respiration and the growth and resistance of plants to stress conditions and phytopathogens [42]. Phenolic compounds are usually excreted by plant roots into the soil and accumulated during the decomposition of plant residues. Consequently, bacterial strains able to degrade aromatic hydrocarbons can be found even in uncontaminated soils. Given this, it is not surprising that strain Ch2 utilized a few aromatic carboxylic acids as growth substrates. The ability of the strain to degrade toxic anthropogenic compounds such as CAP and GP is particularly interesting, because it can help improve understanding of the mechanisms by which new metabolic pathways in bacteria emerge and be interesting in the application of this strain for the cleanup of environmental pollution.

### 3.2. Identification of Ch2 Strain

The 16S rRNA gene sequence analysis was used to identify the Ch2 strain. The phylogenetic analysis of the 1415-bp 16S rRNA gene fragment revealed that this strain belongs to the species *Pseudomonas putida*. The 16S rRNA gene sequence alignment of the isolate showed 100% similarity with the appropriate sequence of the type strain *P. putida* ATCC11172. The nucleotide sequence of the 16S rRNA gene of *P. putida* strain Ch2 was deposited in GenBank with accession number ON203962. 

### 3.3. The Growth Dynamics of P. putida Strain Ch2 in Mineral Medium Supplemented with Caprolactam and Its Intermediates

CAP catabolism in bacteria is a poorly studied process, and genes and enzymes that are involved in the initial steps of this process have not yet been identified. Only one biochemical pathway for caprolactam degradation has been described: caprolactam→6-aminohexanoate→6-oxohexanoate→adipate [12,43]. Further conversion of adipate occurs through the biochemical pathway of fatty acid oxidation (β-oxidation), leading to the formation of succinate and acetyl-CoA that enter the Krebs cycle (Figure S1).

**Figure 1:**
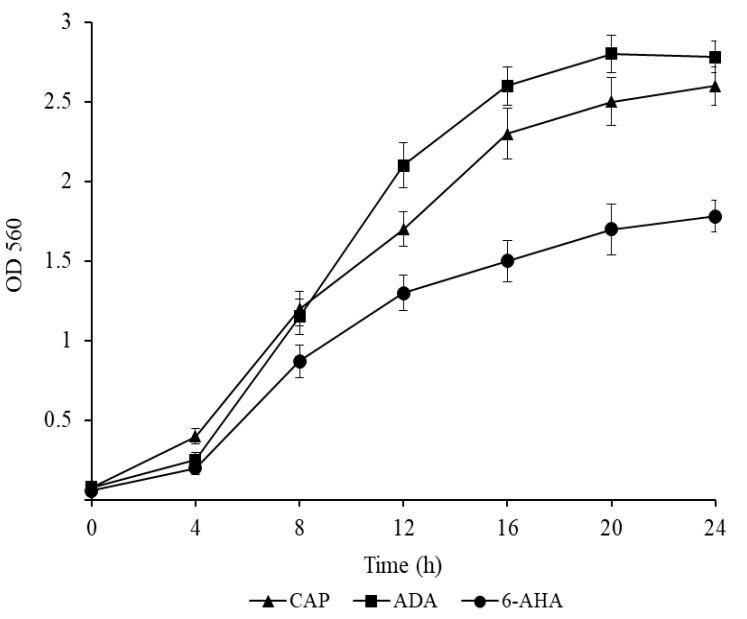
Growth dynamics of *P. putida* strain Ch2 in liquid mineral medium M9 containing caprolactam (CAP), adipic acid (ADA) and 6-aminohexanoic acid (6-AHA) as sole carbon and energy sources. The substrates were used at a concentration of 1.0 g/L. The designation 6-AHA is lost in the legend of the figure. The correct picture was inserted into the cover letter. Please correct.

The following results were obtained: *P. putida* Ch2 grew both in liquid and agar-containing M9 medium supplemented with CAP from 0.5 to 5.0 g/L. The growth of the Ch2 strain was also investigated in M9 with 6-AHA and ADA, the caprolactam pathway intermediates, at a concentration of 1.0 g/L. The strain utilized the said substrates as sole carbon and energy sources, indicating that CAP degradation in *P. putida* Ch2 occurs through the known biochemical pathways (Figure 1).

### 3.4. Genetic Control of Caprolactam and Salicylate Degradation in P. Putida Strain Ch2

It was shown that the ability of bacteria of the genus *Pseudomonas* to utilize CAP is determined by conjugative plasmids (CAP plasmids) that contain the genetic information necessary for the full mineralization of the xenobiotic [12]. Importantly, no plasmid-free *Pseudomonas* strain able to degrade CAP has been described so far. The recent paper [44] reported on the study of the enzymes and genes involved in CAP degradation in *P. jessenii* GO3, but the evidence in support of the localization of catabolic genes on the plasmid DNA was not provided. Based on this fact, we supposed that *P. putida* Ch2 bears the caprolactam biodegradation plasmid. As to salicylate, genes responsible for its oxidation can be localized both on plasmids and chromosomes [45]. Thus, the presence of two catabolic plasmids could not be excluded: one of these plasmids controls the degradation of caprolactam and the second one encodes the degradation of salicylate. Recently, we described SAL/CAP plasmids, a new type of CAP plasmid bearing two catabolic operons [46]. So, it was assumed that the studied strain may contain SAL/CAP plasmid.

**Figure 2:**
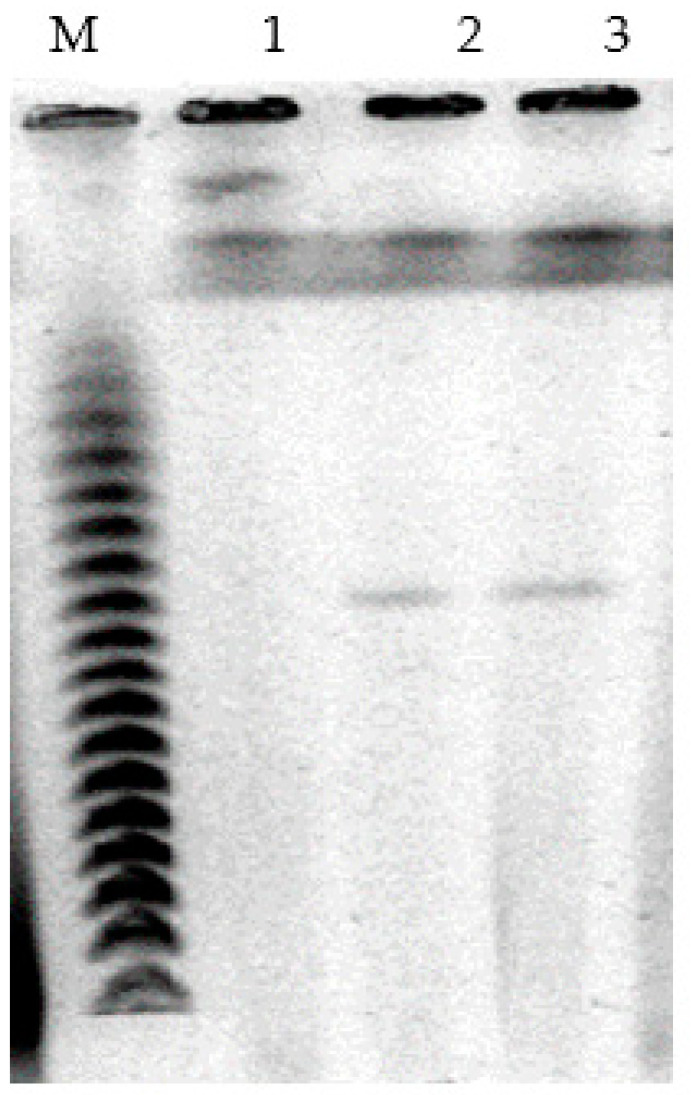
Electropherogram of plasmid DNA of *P. putida* strain Ch2 and *P. putida* KT2442(CAP): M—Lambda Ladder PFG Marker (New England BioLabs), 1—a plasmid-free recipient strain of *P. putida* KT2442, 2—wild-type *P. putida* strain Ch2, 3—a transconjugate strain of *P. putida* KT2442 (CAP).

For visualization of plasmids, genomic DNA pulsed-field gel electrophoresis was applied. The obtained results showed that cells of *P. putida* Ch2 bear megaplasmids that are approximately 550 kb in size. To verify if CAP and/or SAL degradation is determined by a megaplasmid, it was transferred by conjugation to a recipient strain *P. putida* KT2442 (Gfp^+^, Km^r^, Rif^r^). The results showed that transconjugant colonies had grown only on the medium containing CAP, although they did not utilize salicylate. Our multiple attempts to get transconjugants on the medium with salicylate failed. In further analysis, plasmid DNA was detected in CAP^+^ transconjugants, and its size was found to be similar to that of the donor *P. putida* strain Ch2 (Figure 2). Thus, the ability of *P. putida* strain Ch2 to utilize CAP is determined by plasmid genes. As to the genes involved in salicylate degradation, they are much more likely to be located on the chromosome.

### 3.5. The Growth Dynamics and Glyphosate Degradation by P. putida Strain Ch2 during Cultivation in the Liquid Mineral Medium

To study the growth dynamics and GP consumption, the inoculum was prepared in a particular way (see Section 2) to induce the synthesis of enzyme systems involved in GP metabolism and depletion of the intracellular phosphorus pool [47].

**Figure 3:**
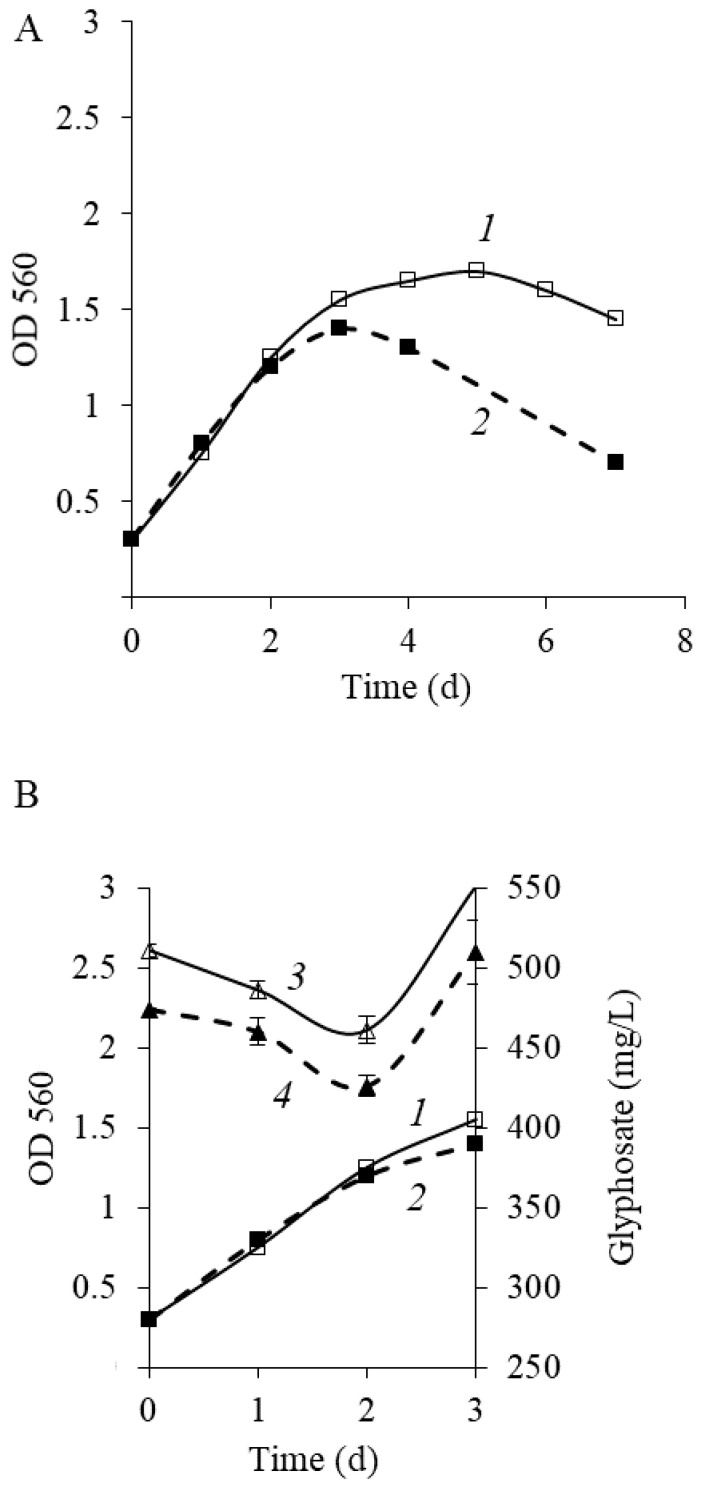
(**A**) Growth dynamics of *P. putida* strain Ch2 in liquid MS1 medium with glyphosate as a sole phosphorus source: 1—the first passage, 2—the second passage. (**B**) The dynamics of glyphosate consumption by *P. putida* strain Ch2: 1—OD, the first passage, 2—OD, the second passage, 3—GP concentration, the first passage, 4—GP concentration, the second passage.

Figure 3 shows that after phosphorus starvation, the maximal value of OD560 of culture in the medium with glyphosate was 1.6 (Figure 3A, curve 1). Notably, an increase in OD occurred proportionately to a reduction in glyphosate level, indicating that *P. putida* strain Ch2 can use glyphosate as a phosphorus source. Intense GP consumption was observed during the active growth of a culture (1–2 days) (Figure 3B, curve 3). In this phase, phosphorus is required for biosynthetic processes including synthesis of nucleic acids, ATP and other nucleoside phosphates, phospholipids and so forth. In parallel to the deceleration of culture growth (after the 2nd day), the peak area rose with a retention time equal to the glyphosate standard. The pattern of glyphosate consumption was similar to that of *Achromobacter* sp. strain Kg 16 through HPLC under the same conditions. The study also explained that it was happened due to the formation of acetylglyphosate, a metabolite of glyphosate [48]. In our study, it has been assumed that the generation of metabolites that can veil the presence of glyphosate in the phase of growth deceleration is also possible.

As reported earlier, the destructive activity of *Ochrobactrum anthropi* GPK3 cells adapted to growing in GP when they were subcultured repeatedly was 1.5–2-fold higher than that of nonadaptive cells [31]. To clarify if *P. putida* Ch2 cells adaptation to GP can contribute to enhanced herbicide consumption, the cells previously grown on GP (the first passage) in the logarithmic phase were added into a fresh medium at a ratio of 1/10 (*v*/*v*) and cultured under the same conditions. The results demonstrated that the adaptive cells of *P. putida* Ch2 after subculture retained their ability to degrade GP, and yet herbicide degradation was not improved (Figure 3, curve 4). Furthermore, in the second passage, during the stationary growth phase, a sharp decrease in optical density was seen in comparison to that of in the first passage. So, optical density value after 7 days of the experiment in the second passage was two times less than that of in the first passage (0.7 and 1.45, respectively) (Figure 3A, curve 2). 

Currently, there are two pathways of GP degradation in bacteria. The first pathway involves direct cleavage of the chemically inert C-P bond by a multicomponent enzyme complex, which is known as the C-P lyase complex. In this case, the formation of sarcosine and inorganic phosphorus (Pi) occurs (C-P lyase pathway) [49]. In the second pathway, an herbicide molecule is initially attacked by an enzyme known as glyphosate oxidoreductase (glyphosate oxidoreductase pathway). This enzyme catalyzes the cleavage of the C-N bond in the glyphosate molecule with subsequent formation of AMPA and glyoxylate in stoichiometric proportion [22,29]. Further, the generated glyoxylate as a suitable energy substrate is then metabolized in the Krebs cycle. In regard to AMPA, in strains able to degrade GP, it is more often not involved in mineralization but excreted into the external medium [49,50] (Figure S2). 

**Figure 4:**
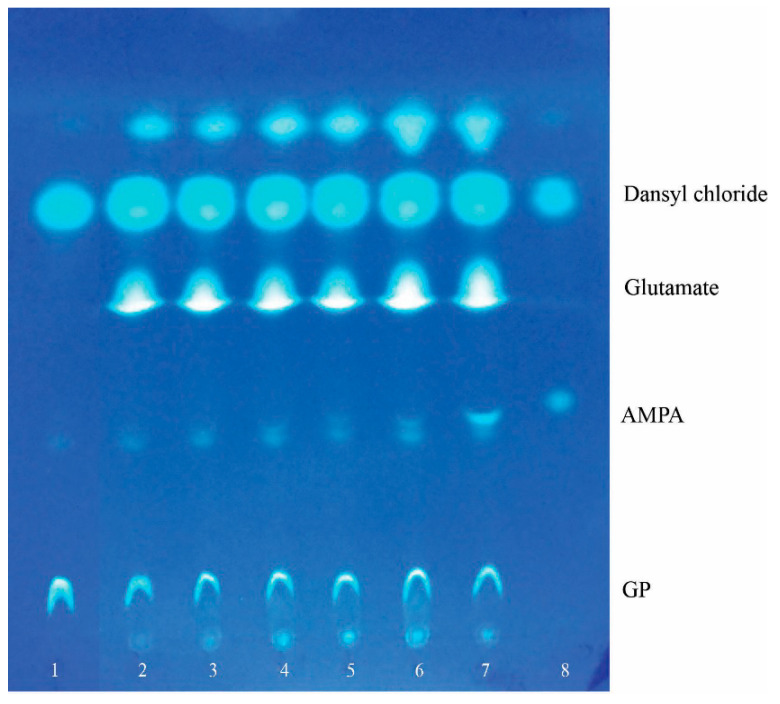
Mobility of dansyl derivatives of glyphosate and its transformation products that are present in the culture liquid of *P. putida* strain Ch2: 1—GP, 2–6—the culture liquid; 2—start point, 3—the 1st day of cultivation, 4—the 4th day of cultivation, 5—the 7th day of cultivation, 6—the 13th day of cultivation, 7—the 13th day of cultivation + AMPA as internal standard, 8—AMPA.

To determine through what biochemical pathway glyphosate degradation occurs in *P. putida* strain Ch2, we analyzed metabolites accumulated in culture liquid when cells were grown on MS1 medium with GP. For this analysis, thin-layer chromatography was used and allowed for identification of AMPA, one of the mentioned GP transformation products (Figure 4). It is important to emphasize that AMPA was detected in culture liquid only in the deceleration growth phase and stationary phase when cell division and phosphorus consumption were ceased (Figure 4, track 5, 6). Therefore, the presence of this metabolite in culture liquid might tell us that the C-N bond of glyphosate is the primary site of attack.

### 3.6. The Effect of Glyphosate on Cell Morphology in P. putida Strain Ch2

**Figure 5:**
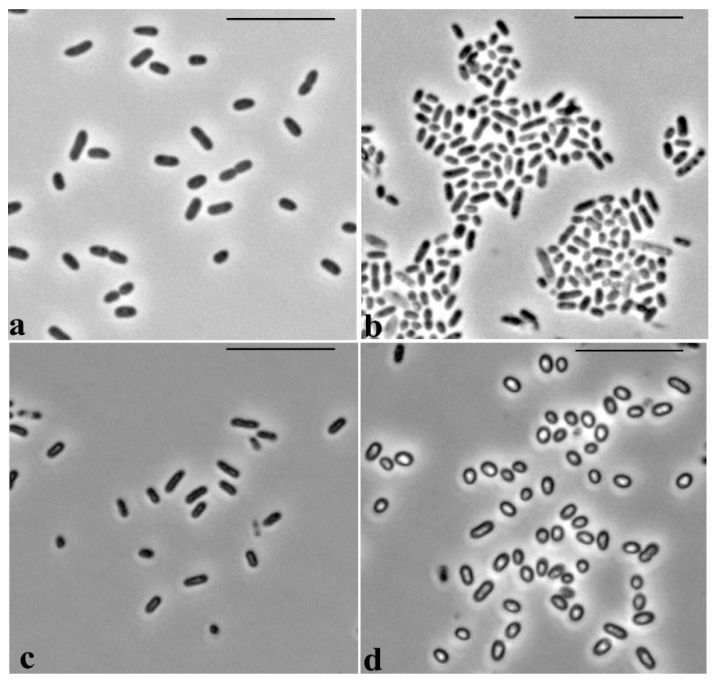
Phase contrast microscopy of *P. putida* Ch2 cells. (**a**,**b**) LB medium: (**a**) the 1st day of growth; (**b**) the 4th day of growth. (**c**,**d**) MS1 mineral medium with glyphosate: (**c**) the 1st day of growth, (**d**) the 4th day of growth. Bar = 10 µm.

Microscopic observations revealed specific morphological features of the cells of *P. putida* Ch2 during growth on LB medium and mineral MS1 medium supplemented with GP. Cells grown on LB medium are represented by rod-shaped forms with sizes of 1.8–2.3 × 0.8–0.9 µm (Figure 5a). In the inoculum prepared in MS1 medium, the cells had similar rod-shaped forms and dimensions, 1.8–2.0 × 0.6–0.65 µm, and their size did not alter for the first days of growth in MS1 medium with GP (Figure 5c). However, after 4 days, the cells in this medium became rounded, lost their rod-like shape, and became refractory, shorter and considerably increased in width: 1.0–1.5 × 1.0–1.2 µm (Figure 5d).

**Figure 6:**
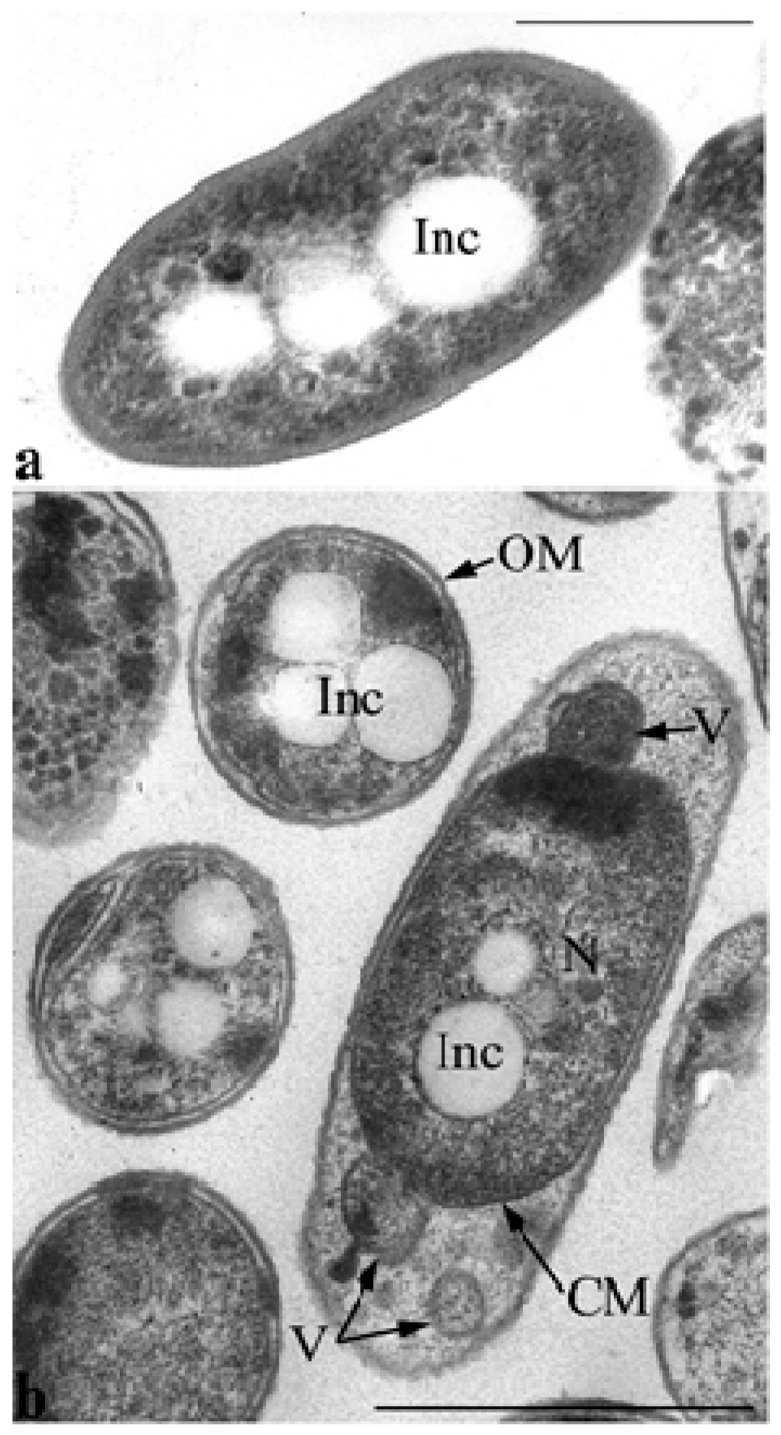
Transmission electron microscopy. (**a**) Ultrathin sections of *P. putida* Ch2 cells grown on LB agarized medium and (**b**) in MS1 medium with glyphosate as the sole source of phosphorus (inoculum). Designations: OM—outer membrane; CM—cytoplasmic membrane; V—vesicle; Inc—PHA inclusions; N—nucleoid. Bar =1 µm.

Electron microscopy of ultrathin sections revealed that ultrastructural organization of the inoculum cells grown in MS1 medium with GP differed markedly from the control cells grown in LB (Figure 6). Thus, inoculum cells are characterized by enlarged periplasmic space at the poles into which the vesicles of the cytoplasmic membrane with the components of the cytoplasm are released by budding (Figure 6b).

It is noteworthy that a particular feature of *P. putida* strain Ch2 is its ability to form polyhydroxyalkanoate inclusions that are present in different amounts in the cell’s cytoplasm during growth on different media. Figure 6a shows sections of *P. putida* Ch2 cells grown on agarized LB medium for 4 days. PHAs under these conditions (the culture is in the stationary phase of growth) are detected mainly in the form of accumulations of small- and medium-sized electron-transparent spherical inclusions in the cytoplasm of cells. In some cells in this phase of growth, such inclusions are not found, and rarely, in some cells, they fill almost the entire volume of the cytoplasm. PHA inclusions are also present in the cells of the inoculum (Figure 6b).

**Figure 7:**
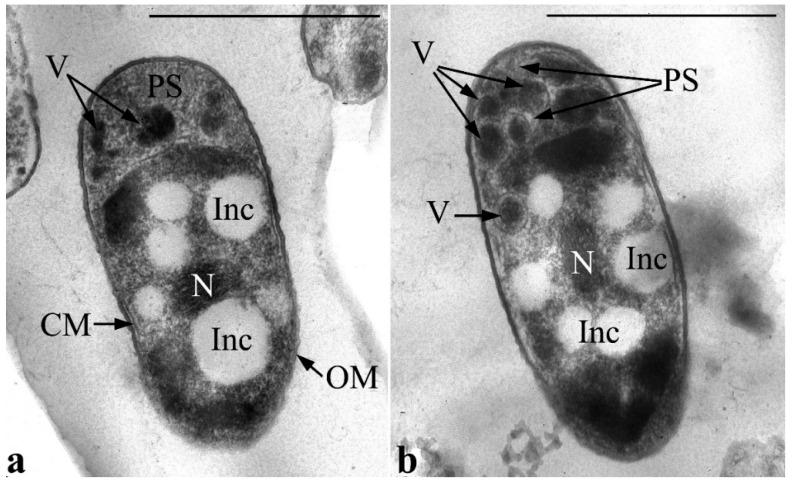
Transmission electron microscopy. Ultrathin sections of *P. putida* Ch2 cells during growth in a liquid MS1 medium with glyphosate, the 1st day (**a**,**b**). Designations: OM—outer membrane; CM—cytoplasmic membrane; V—vesicles; Inc—PHA inclusions; N—nucleoid; PS—periplasmic space. Bar = 1 µm.

During the cultivation of the strain in MS1 medium with GP, alterations in the ultrathin organization of the cell accumulate over time. So, after one day of culture growth, the number of vesicles has increased in large periplasmic areas. Vesicles are distinguished by high electron density, and the density of the cells’ periplasmic space itself also increases (Figure 7). This can presumably imply elevated phosphorus levels in matrix vesicles and, probably, their active involvement in metabolism of GP (specifically, in the primary stage of this metabolic process). Importantly, at this stage, the nucleoid is usually found condensed in the form of tightly packed fibers in the center of the cell cytoplasm. The condensed state of the nucleoid in active growing cells might be associated with protection against aggressive intermediates of primary degradation of GP. 

**Figure 8:**
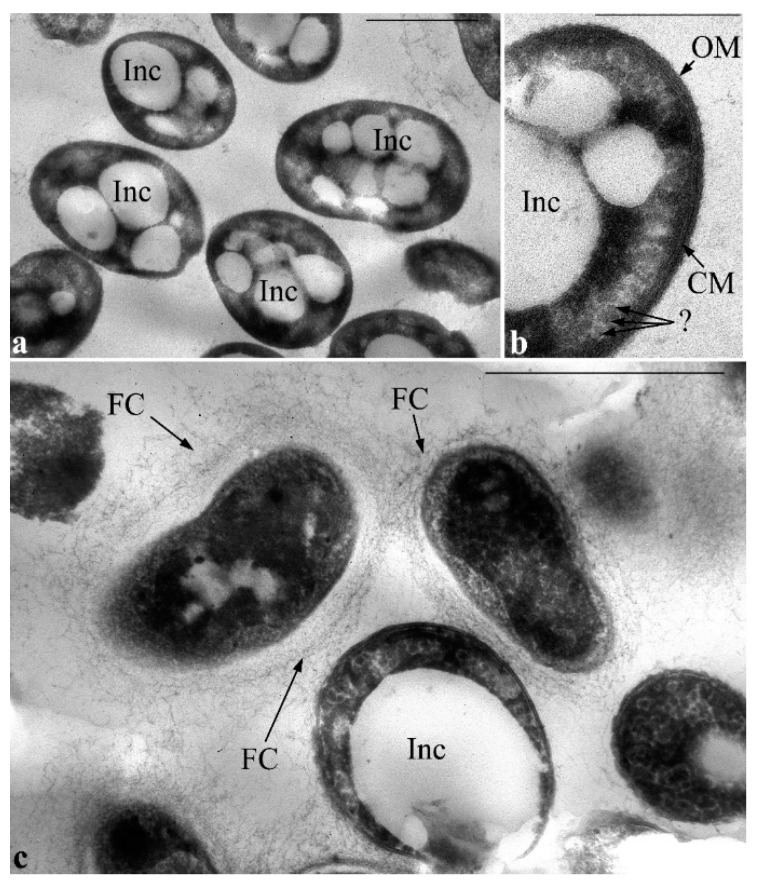
Transmission electron microscopy. Ultrathin sections of *P. putida* Ch 2 cells grown in a liquid MS1 medium with glyphosate: the 4th day of growth (**a**,**b**) and 8th day of growth (**c**). Designations: OM—outer membrane; CM—cytoplasmic membrane; Inc—PHA inclusions; FC—fibrillar cover. Mark “?” in image (**b**)—inclusions of an unknown nature are indicated, resembling high-molecular starch-like compounds. Bar = 1 µm.

Later (on the 4th day of culture growth), clusters of vesicles were not viewed in extended periplasmic areas, meanwhile, the amount and size of PHA inclusions drastically increased; they filled almost the entire volume of cell cytoplasm. Nevertheless, the cells are characterized by the integrity of all the envelope elements. Also, in the cytoplasm of cells under these conditions, there are inclusions that have some similarity with inclusions of starch (Figure 8b, indicated by a question mark).

After 8 days of cultivation, some of the cells that contain very large PHA inclusions are lysed, most likely, because of mechanical disruption of cell envelope. Most cells in the cell population transform into the resting state. PHA inclusions are virtually absent in the cytoplasm of these cells, their cytoplasmic content appears denser and some extracellular material such as fibrillar layers in the form of a capsule is visualized; it is not characteristic of vegetative cells (Figure 8c).

**Figure 9:**
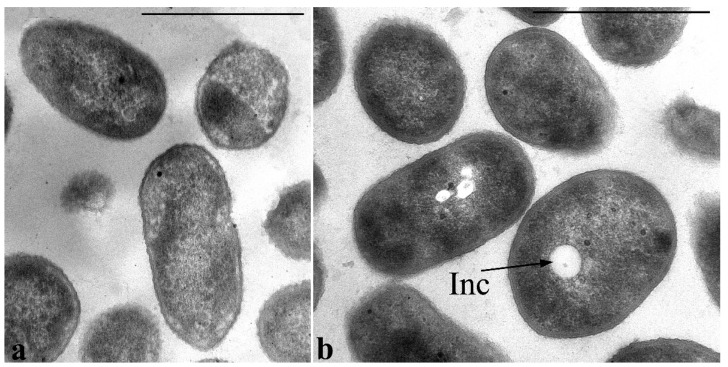
Transmission electron microscopy. Ultrathin sections of *P. putida* Ch 2 cells grown in an MS1 medium supplemented with caprolactam (**a**) and salicylate (**b**). Single small electron-transparent inclusions are rarely found in the cytoplasm of cells grown on salicylate (**b**). Designations: electron-transparent inclusions: Inc. Bar = 1 µm.

During cultivation of *P. putida* Ch2 in MS1 media containing caprolactam or salicylate, on rare occasions, single small electron-transparent PHA inclusions in the cell cytoplasm can be detected (Figure 9). In these variants a nucleoid does not fold into compact structures and is evenly distributed in the center of the cytoplasm. The nucleoid is seen as a less dense region with granular-fibrillar structure in the cytoplasm that is similar to that viewed during cultivation of the strain on standard growth media.

Thus, bacterial growth in the presence of GP, even at the early stage of its degradation, is accompanied by a unique substrate-dependent structural reorganization of the cell’s cytoplasm. Cells under these conditions are most likely compartmentalizing their cytoplasm by forming cytoplasmic membrane vesicles containing specific electron-dense content. It is supposed that these membrane formations are a kind of analogue of metabolosomes, in which the process of primary degradation of the herbicide can take place. The cells carry out the output of these vesicular formations to periplasmic space, thereby separating the process of utilization of toxicants from the main volume of the cytoplasm, and mainly from nucleoids.

In this work, it is the first time when accumulation of PHA inclusions is shown during growth of *P. putida* Ch2 on the mineral medium containing GP as a sole source of phosphorus. It is known that accumulation of PHAs in the cells helps bacteria to survive under stress conditions such as extreme temperatures, dehydration, osmotic and oxidative stress and the presence of heavy metals and other recalcitrant organic compounds [51]. Bacterial strains of the genus *Pseudomonas* have been described as being capable of synthesizing PHAs during the degradation of toxic organic compounds such as phenol [18], terephthalic acid [16], naphthalene and other mono- and poly-aromatic hydrocarbons [17]. Apparently, synthesis of the excess amount of PHAs in *P. putida* strain Ch2 is a protective cell response to stress conditions during cultivation in GP-containing medium.

## 4. Conclusions

In the present study, a novel *P. putida* strain, Ch2, with a unique ability to degrade anthropogenic compounds (CAP as the sole carbon and energy source and GP as a source of phosphorus), has been isolated and characterized. The reported degradative characteristics of the strain may be of both scientific and practical interest for bioremediation efforts. For the first time, the strain was shown to accumulate PHA inclusions during growth on the mineral medium containing GP. This peculiarity has opened wider prospects for its technological application. The performing of a complete genome analysis will contribute to a better understanding of the decomposition mechanisms of the tested toxic compounds and PHAs production in *P. putida* Ch2.

## Data Availability

Data are contained within the article.

## Supplementary Materials

The following supporting information can be downloaded at: https://www.mdpi.com/article/10.3390/microorganisms11030650/s1. Figure S1. Principal *epsilon*-caprolactam biodegradation pathway in bacteria. Figure S2. Principal glyphosate biodegradation pathways in bacteria.
