# Supplementary material for: Characterization of a New Pseudomonas Putida Strain Ch2, a Degrader of Toxic Anthropogenic Compounds Epsilon-Caprolactam and Glyphosate
Source: Microorganisms. 2023 Mar 3;11(3):650. doi: 10.3390/microorganisms11030650 (PMC10053300; doi:10.3390/microorganisms11030650)
Supplement: Supplementary file 1 [file microorganisms-11-00650-s001.zip › microorganisms-2235099-supplementary.pdf]

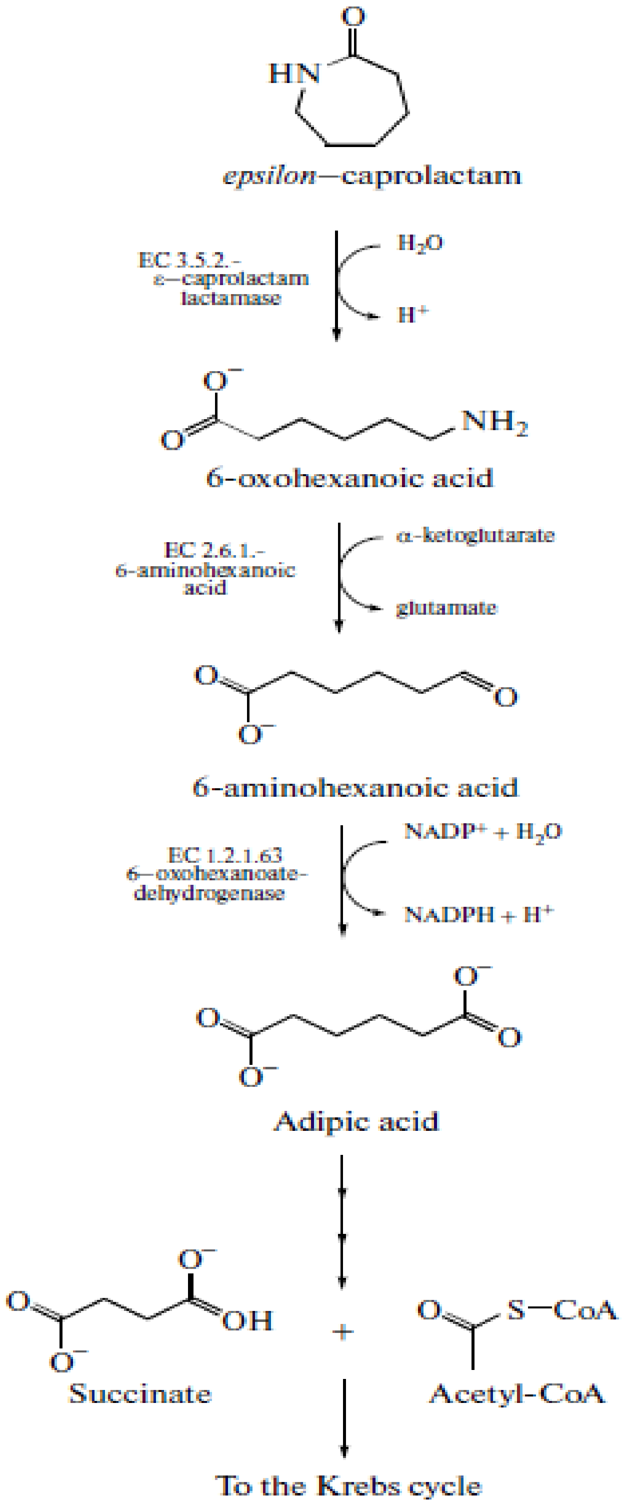


**Figure S1**. Principal *epsilon*-caprolactam biodegradation pathway in bacteria [43].

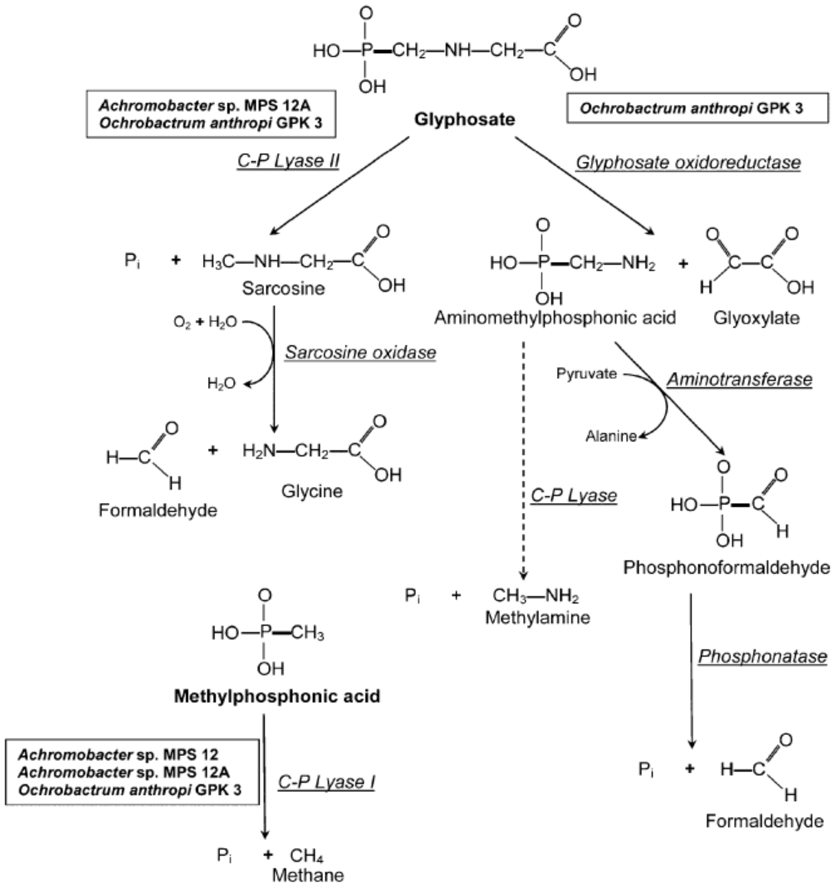


**Figure S2**. Principal glyphosate biodegradation pathways in bacteria [50].
